# NPRL2 restricts porcine reproductive and respiratory syndrome virus replication by targeting viral Nsp1α for autophagic degradation

**DOI:** 10.1186/s13567-026-01717-x

**Published:** 2026-02-25

**Authors:** Heyou Yi, Shaojun Wang, Qiumei Wang, Lechen Lu, Ermin Xie, Ruirui Ye, Heng Wang, Guihong Zhang

**Affiliations:** 1 Key Laboratory of Animal Pathogen Infection and Immunology of Fujian Province, College of Animal Sciences, Fujian Agriculture and Forestry University, Fuzhou, 350002 China; 2 Guangdong Provincial Key Laboratory of Zoonosis Prevention and Control, College of Veterinary Medicine, South China Agricultural University, Guangzhou, 510462 China

**Keywords:** NPRL2, PRRSV, Nsp1α, autophagic degradation, host restriction factor

## Abstract

Host factors that directly target viral immune antagonists are crucial for antiviral defense. In this study, we identify NPRL2 as a novel host restriction factor that directly interacts with the porcine reproductive and respiratory syndrome virus (PRRSV) protein Nsp1α, a key viral virulence factor. This interaction is mediated by the C-terminal domain of NPRL2 and the PCPα domain of Nsp1α. Functional studies demonstrated that NPRL2 overexpression inhibits PRRSV replication, while its knockdown enhanced viral propagation. Mechanistically, NPRL2 acts as a bridge, mediating K63-linked ubiquitination of Nsp1α at lysine 150 and subsequently recruiting the autophagic machinery for its degradation. This process was confirmed by monitoring LC3-II conversion and autophagic flux. Our findings reveal a precise mechanism by which NPRL2 antagonizes PRRSV by targeting a critical viral protein for autophagic degradation, highlighting the therapeutic potential of harnessing the host’s ubiquitin–autophagy pathway to combat viral infections.

## Introduction

Porcine reproductive and respiratory syndrome (PRRS), caused by the PRRS virus (PRRSV), is among the most economically devastating diseases in the global swine industry, primarily characterized by reproductive failure in sows and respiratory distress in piglets [1, 2]. PRRSV is an enveloped, single-stranded positive-sense RNA virus of the *Arteriviridae* family [3]. The virus’s high genetic diversity and sophisticated immune evasion strategies have consistently challenged the development of broadly effective vaccines or antiviral drugs [4–6]. A deeper understanding of the virus–host interactions is therefore crucial for identifying new therapeutic targets.

A central player in PRRSV immune evasion is the Nsp1α. As an early-expressed protein, Nsp1α acts as a key virulence factor that disrupts the host innate immune response, notably by suppressing type I interferon (IFN) production through multiple mechanisms [7–12]. While these proviral functions of Nsp1α are well-appreciated, the mechanism of host antagonizing this protein is still unclear. Identifying host factors that directly target and neutralize Nsp1α may reveal new principles of antiviral defense.

Among the host’s arsenal of antiviral defenses, selective autophagy has emerged as a critical mechanism for the direct elimination of intracellular pathogens [13, 14]. This process relies on the precise tagging of target proteins with specific ubiquitin chains. In particular, K63-linked polyubiquitin chains often serve as a definitive signal for cargo recognition by autophagic receptors, leading to its sequestration into autophagosomes and subsequent lysosomal degradation [15, 16]. Therefore, identifying the E3 ubiquitin ligases or adaptor proteins that mediate the ubiquitination of viral proteins is a central question in antiviral immunity.

NPRL2 (Nitrogen Permease Regulator 2-like), also known as tumor suppressor candidate 4 (TUSC4), is a potential tumor suppressor gene implicated in antiproliferative effects and cancer pathogenesis [17, 18]. Beyond its role in oncogenesis, emerging evidence has begun to illuminate the capacity of NPRL2 to directly orchestrate protein degradation. A pivotal recent study demonstrated that NPRL2 promotes the TRIM16-mediated ubiquitination and degradation of Galectin-3 to modulate cuproptosis in T cells [19], thereby establishing NPRL2 as a key regulator of protein stability in host immune cells.

However, a fundamental question remains unanswered: can NPRL2 employ this protein-targeting function as a direct antiviral mechanism? Its potential to recognize and facilitate the degradation of viral proteins, thereby executing intrinsic antiviral defense, remains unexplored. This critical gap in knowledge presents an intriguing hypothesis: could NPRL2 function as a broad-spectrum host factor that bridges viral antagonists to the cellular degradation machinery to restrict viral infection?

In this study, we identify NPRL2 as a novel host restriction factor that directly binds to PRRSV Nsp1α. We further elucidate the mechanism by which NPRL2 facilitates the K63-linked ubiquitination of Nsp1α at lysine 150 (K150), marking it for recognition and degradation via the autophagy–lysosome pathway. This process significantly inhibits PRRSV replication. Our work not only reveals a previously unknown host defense mechanism but also suggests a potential strategy for developing therapies aimed at the targeted degradation of viral proteins.

## Materials and methods

### Virus and cells

HEK-293 T cells and Marc-145 cells were cultured in Dulbecco’s modified Eagle’s medium (DMEM; Gibco, USA) supplemented with 10% fetal bovine serum (FBS; VivaCell, C04001) at 37 °C with 5% CO_2_. PRRSV strain XHGD was propagated in Marc-145 cells and titrated as previously described [20].

### Antibodies and reagents

All antibodies and reagents were obtained from commercial sources as follows. From Cell Signaling Technology: rabbit anti-HA tag (3724). From Proteintech: rabbit anti-Myc (16286-1-AP) and mouse anti-β-actin (66009-1-Ig). From Beyotime: mouse anti-Flag (AF519), mouse anti-HA (AH158), rabbit anti-GST tag (AG768), GST tag purification resin (P2230), mouse IgG (A7028), rabbit IgG (A7016), and Lipo8000 Transfection Reagent (C0533). From TransGen Biotech: mouse anti-GAPDH (HC301-01). From Abmart: rabbit anti-Flag (T20008). From Santa Cruz Biotechnology: rabbit anti-NPRL2 (sc-376986). From Jinnuo Baitai Biotechnology: PRRSV N protein antibody (JN0401). From GeneTex: PRRSV Nsp1α antibody (GTX133695). From Invitrogen: Alexa Fluor 594 goat anti-rabbit IgG (A11012), Alexa Fluor 488 goat anti-mouse IgG (A11011), Alexa Fluor 647 goat anti-mouse IgG (A21235), and Alexa Fluor 647 goat anti-rabbit IgG (A21244). From Abcam: rabbit anti-LC3B (ab192890), rabbit anti-LAMP1 (ab278043), and donkey anti-mouse IgG H&L (Alexa Fluor 405) (ab175658). From Absolute Biotech, mouse anti-dsRNA (J2 clone) (10010500). From LI-COR: IRDye 800CW goat anti-mouse IgG (926-32210) and IRDye 800CW goat anti-rabbit IgG (926-32211). The inhibitors 3-methyladenine (3-MA; HY-19312) and MG132 (HY-13259) were purchased from MedChemExpress.

### Plasmids and siRNAs

Full-length cDNAs of PRRSV Nsp1α was amplified from the PRRSV genome and cloned using ClonExpress Ultra One Step Cloning Kit (C115, Vazyme) into the respective pCAGGS-Flag, EGFP-C1 and pGEX-4 T-2-N vectors to generate the plasmids Flag-Nsp1α, EGFP-Nsp1α and GST-Nsp1α, respectively. The full-length cDNA of NPRL2 was amplified from porcine alveolar macrophages (PAMs) and inserted into pCAGGS-HA and pCAGGS-Myc vectors to produce HA-NPRL2 and Myc-NPRL2 expression constructs. We generated a series of truncated mutants of Flag-Nsp1α and HA-NPRL2 using PCR-based cloning. Using gene synthesis, three individual single-point mutants of Nsp1α (K117A, K150A, and K169A) were generated.

A series of ubiquitin mutant plasmids—pRK5-HA-UbK6, pRK5-HA-UbK11, pRK5-HA-UbK27, pRK5-HA-UbK29, pRK5-HA-UbK33, pRK5-HA-UbK48, and pRK5-HA-UbK63—in which all lysine residues except one (the indicated position) were mutated to arginine, along with wild-type HA-tagged ubiquitin, were maintained in our laboratory.

We used an siRNA targeting *NPRL2*, as previously reported [21]. Marc-145 cells were transfected with 20 nM siRNA targeting *NPRL2* or a negative control siRNA using Rfect siRNA/miRNA Transfection Reagent (11013, Baidai biotechnology) according to the manufacturer’s instructions. The siRNA sequence is complementary to the monkey *NPRL2* gene, and the coding strand sequence is 5′-AUAAGCCCGAACUGGAUCAGCUUCC-3′.

### Virus infection and titration

Marc-145 cells were seeded in 6-well plates and infected with PRRSV at a multiplicity of infection (MOI) of 0.1 for 1 h at 37 °C. The inoculum was then removed, and cells were overlaid with fresh maintenance medium (DMEM containing 2% FBS). At the indicated time points post-infection, culture supernatants were collected and serially diluted 10-fold. Each dilution was added to fresh monolayers of Marc-145 cells in 96-well plates (*n* = 8 per dilution). After 1 h of adsorption, the inoculum was replaced with fresh medium. Viral cytopathic effect (CPE) was observed and recorded at 72 h post-infection. The 50% tissue culture infectious dose (TCID_50_) was calculated using the Reed–Muench method.

### Confocal microscopy

Marc-145 cells were fixed with 4% paraformaldehyde for 15 min, permeabilized with 0.1% Triton X-100 for 15 min, and blocked with 5% skim milk in phosphate buffered saline (PBS) for 1 h at room temperature. Cells were then incubated with primary antibodies overnight at 4 °C, followed by incubation with fluorescence-conjugated secondary antibodies for 1 h at room temperature. After washing, nuclei were counterstained with 4′,6-diamidino-2-phenylindole (DAPI). Coverslips were mounted onto glass slides using Antifade Mounting Medium (P0126, Beyotime). Images were acquired using a Leica STELLARIS 8 FALCON confocal microscope.

### Immunoelectron microscopy

Marc-145 cells cotransfected with HA-NPRL2 and Flag-Nsp1α for 24 h were fixed with a mixture of 4% paraformaldehyde and 0.1% glutaraldehyde. Ultrathin cryosections were prepared and subjected to immunogold labeling. Sections were incubated with primary antibodies against Flag (Beyotime, AF519) and HA (CST, 3724) overnight at 4 °C, followed by incubation with corresponding secondary antibodies conjugated to 4-nm (anti-mouse) and 12-nm (anti-rabbit) colloidal gold particles. Samples were examined under a JEM-2010HR transmission electron microscope.

### GST pull-down assay

The pGEX-Nsp1α plasmid was transformed into BL21(DE3) competent cells, and protein expression was induced with isopropyl β-D-1-thiogalactopyranoside (IPTG, ST098, Beyotime). A GST pull-down assay was performed using a GST-tagged protein purification kit (P2262, Beyotime) according to the manufacturer’s instructions. Briefly, GST-Nsp1α fusion proteins were bound to glutathione-Sepharose beads and incubated with lysates of HEK-293 T cells expressing HA-NPRL2 at 4 °C for 3 h. The beads were then thoroughly washed, and bound proteins were eluted for western blot analysis.

### Western blotting and Coimmunoprecipitation (Co-IP)

Cells were lysed with NP-40 lysis buffer (P0013F, Beyotime) supplemented with protease and phosphatase inhibitors. Western blotting was performed as previously described [20]. For the Co-IP assay, cell lysates were incubated with specific antibodies or control IgG overnight at 4 °C with gentle rotation. Protein A/G agarose beads (sc-2003, Santa Cruz Biotechnology) were then added and incubated for 6 h at 4 °C. The immunoprecipitated complexes were washed three times with cold Tris-buffered saline with Tween 20 (TBST) and subsequently analyzed by immunoblotting.

### Statistical analysis

Statistical analyses were performed using GraphPad Prism software (version 7.0). Data are presented as the mean ± SD from at least three independent experiments. Differences between groups were evaluated using an unpaired Student’s *t*-test. Significance levels are denoted as follows: * *P* < 0.05; ** *P* < 0.01; *** *P* < 0.001; **** *P* < 0.0001; not significant (ns), *P* > 0.05.

## Results

### NPRL2 interacted with PRRSV Nsp1α

**Figure 1:**
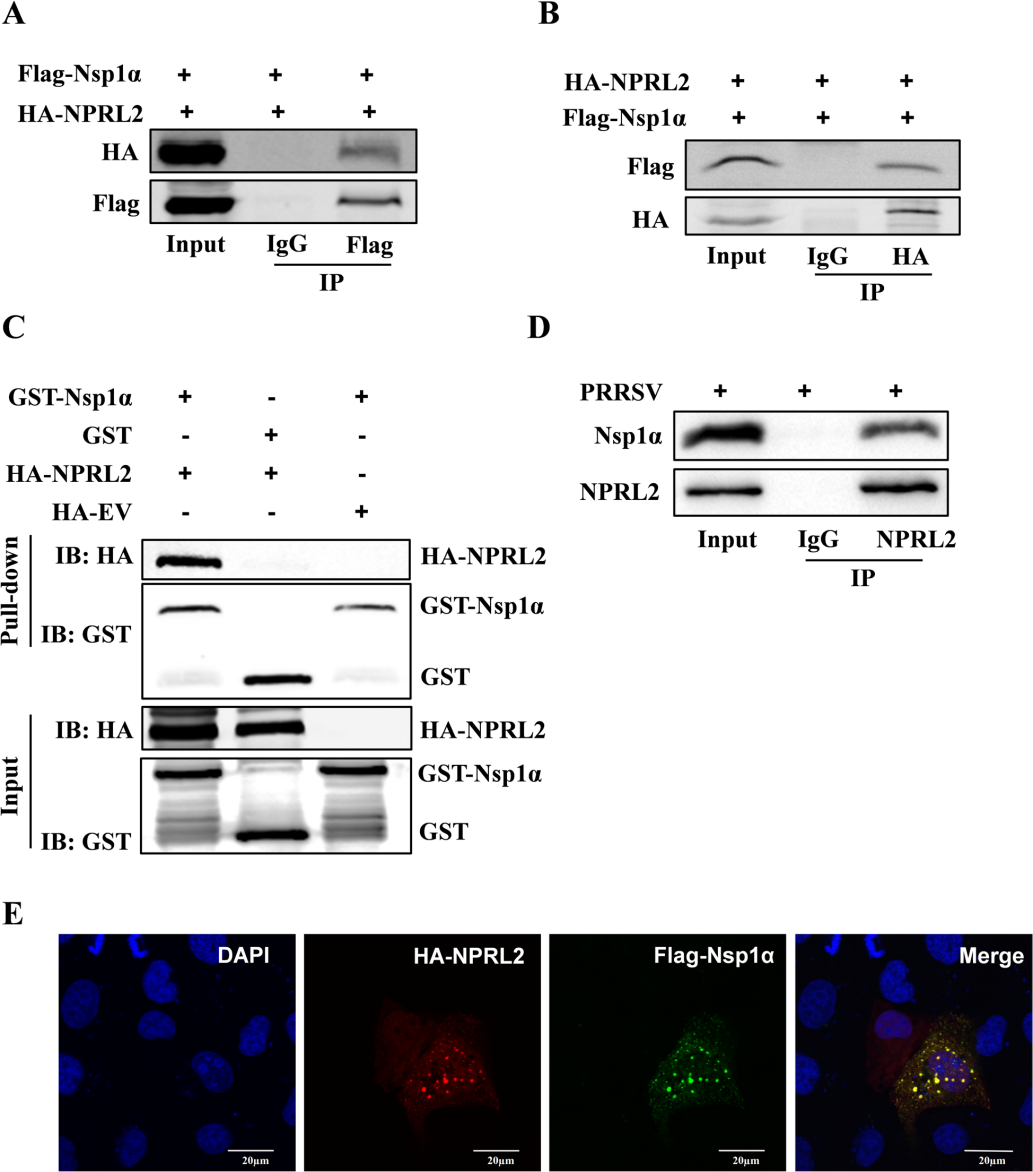
**NPRL2 interacts with PRRSV Nsp1α**. **A**, **B** Coimmunoprecipitation (Co-IP) of NPRL2 and Nsp1α. Lysates from HEK-293 T cells coexpressing HA-NPRL2 and Flag-Nsp1α for 36 h were immunoprecipitated with an anti-Flag (**A**) or anti-HA (**B**) antibody, followed by immunoblotting (IB) with the indicated antibodies. **C** GST pull-down assay demonstrates a direct interaction. Lysates from cells expressing HA-NPRL2 were incubated with immobilized GST or GST-Nsp1α. Bound proteins were analyzed by IB with an anti-HA antibody. The bottom panel shows the input GST fusion proteins probed with an anti-GST antibody. **D** Endogenous NPRL2–Nsp1α interaction upon PRRSV infection. Marc-145 cells were infected with PRRSV (MOI of 1 TCID_50_ per cell) for 24 h. Cell lysates were subjected to Co-IP using antibodies against endogenous NPRL2, followed by IB with the indicated antibodies. IgG was used as an isotype control for immunoprecipitation. **E** Colocalization of NPRL2 and Nsp1α. Marc-145 cells expressing Flag-Nsp1α (green) and HA-NPRL2 (red) were fixed at 24 h post-transfection and immunostained with the indicated antibodies. Nuclei were stained with DAPI (blue). Scale bar, 20 μm.

We previously identified an interaction between NPRL2 and Nsp1α in a yeast two-hybrid screen [20]. To confirm this interaction, we performed coimmunoprecipitation (Co-IP) assays in HEK-293 T cells cotransfected with HA-NPRL2 and Flag-Nsp1α. Immunoprecipitation with either an anti-Flag or anti-HA antibody successfully precipitated the binding partner (Figures 1A and B), confirming a specific interaction. To determine if this binding was direct, we conducted a GST pull-down assay. The results showed that NPRL2 bound directly to GST-Nsp1α, but not to the GST control (Figure 1C). To investigate whether this interaction occurs under physiological conditions, we performed Co-IP assays using lysates from PRRSV-infected Marc-145 cells, which express endogenous levels of both proteins. Immunoprecipitation of endogenous NPRL2 efficiently coprecipitated endogenous Nsp1α from infected cell lysates (Figure 1D). Furthermore, confocal microscopy revealed that NPRL2 and Nsp1α colocalized in both the cytoplasm and the nucleus (Figure 1E).

### Key domain for the interaction between NPRL2 and Nsp1α

**Figure 2:**
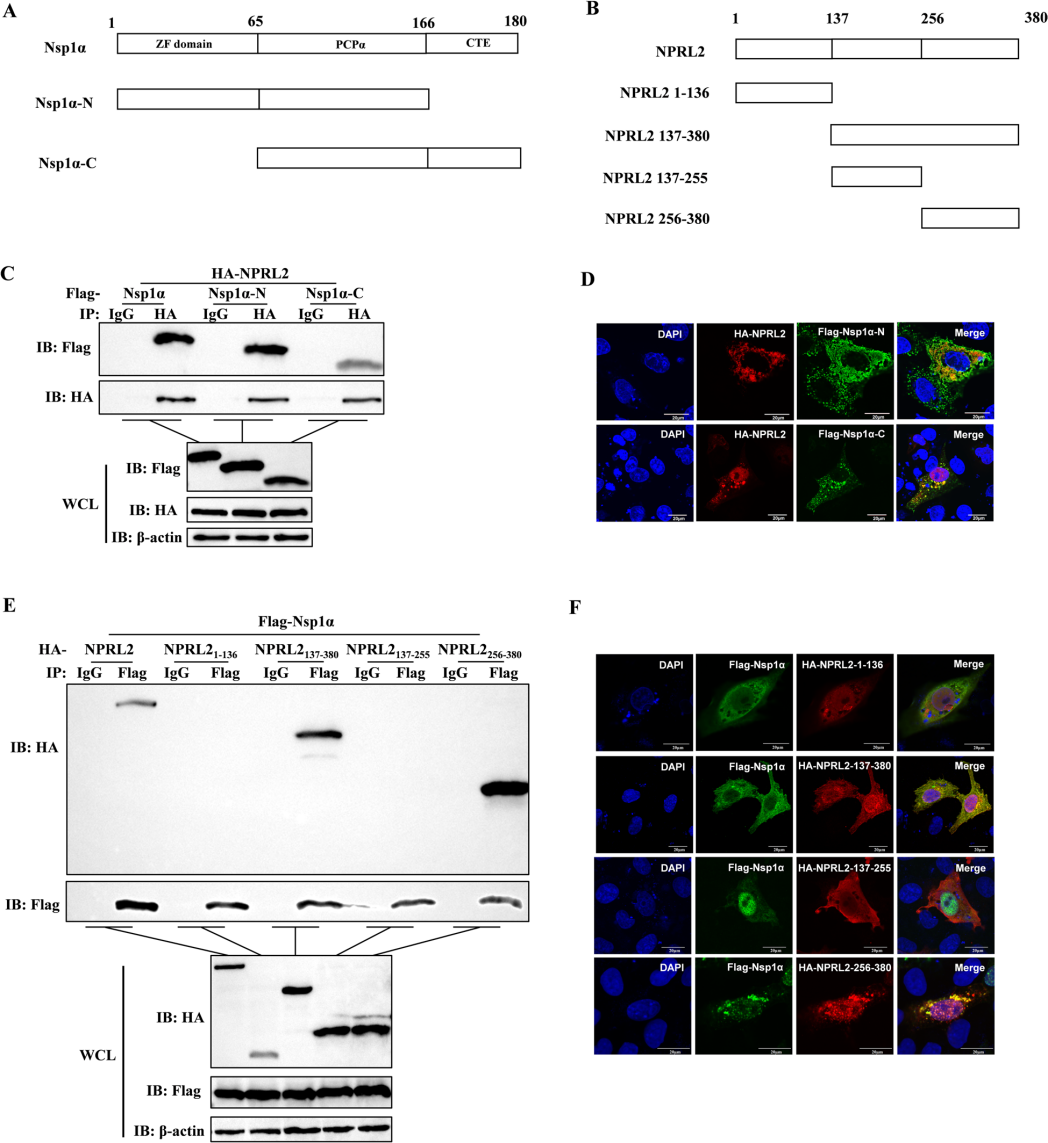
**Identification of critical interaction domains between Nsp1α and NPRL2**. **A**, **B** Schematic diagrams of Nsp1α (A) and NPRL2 (B) deletion mutants. **C** Co-IP analysis of Nsp1α truncations. HEK-293 T cells coexpressing HA-NPRL2 and Flag-tagged Nsp1α (full-length or truncated) were lysed at 36 h post-transfection. The lysates were then subjected to immunoprecipitation separately with anti-HA antibody and control IgG antibody, followed by immunoblotting with anti-Flag, anti-HA, and anti-β-actin antibodies. **D** Colocalization analysis of Nsp1α truncations with NPRL2. Marc-145 cells cotransfected with HA-NPRL2 and Flag-tagged Nsp1α truncations were fixed at 24 h post-transfection. Cells were immunostained with anti-Flag and anti-HA antibodies. Nuclei were stained with DAPI. Scale bar, 20 μm. **E** Co-IP analysis of NPRL2 truncations. Experiments were performed as in (C), but cells were cotransfected with Flag-Nsp1α and HA-tagged NPRL2 (full-length or truncated). Lysates were immunoprecipitated with anti-Flag or control IgG antibody, respectively, followed by immunoblotting. **F** Colocalization analysis of NPRL2 truncations with Nsp1α. Analysis was performed as in (D) for cells expressing Flag-Nsp1α and HA-tagged NPRL2 truncations.

To map the domains mediating the NPRL2–Nsp1α interaction, we generated truncated HA-NPRL2 and Flag-Nsp1α mutants (Figure 2A and B). Co-IP and confocal microscopy analyses revealed that Nsp1α fragments spanning residues 1–166 and 66–180 interacted with NPRL2 (Figure 2C and D), indicating that its PCPα domain (residues 66–166) is critical for binding. Furthermore, NPRL2 truncations containing residues 137–380 or 256–380 interacted with Nsp1α, while fragments 1–136 and 137–255 did not show interaction (Figure 2E and F). Collectively, these results demonstrate that the C-terminal region (residues 256–380) of NPRL2 mediates its association with Nsp1α.

### NPRL2 inhibited PRRSV replication

**Figure 3:**
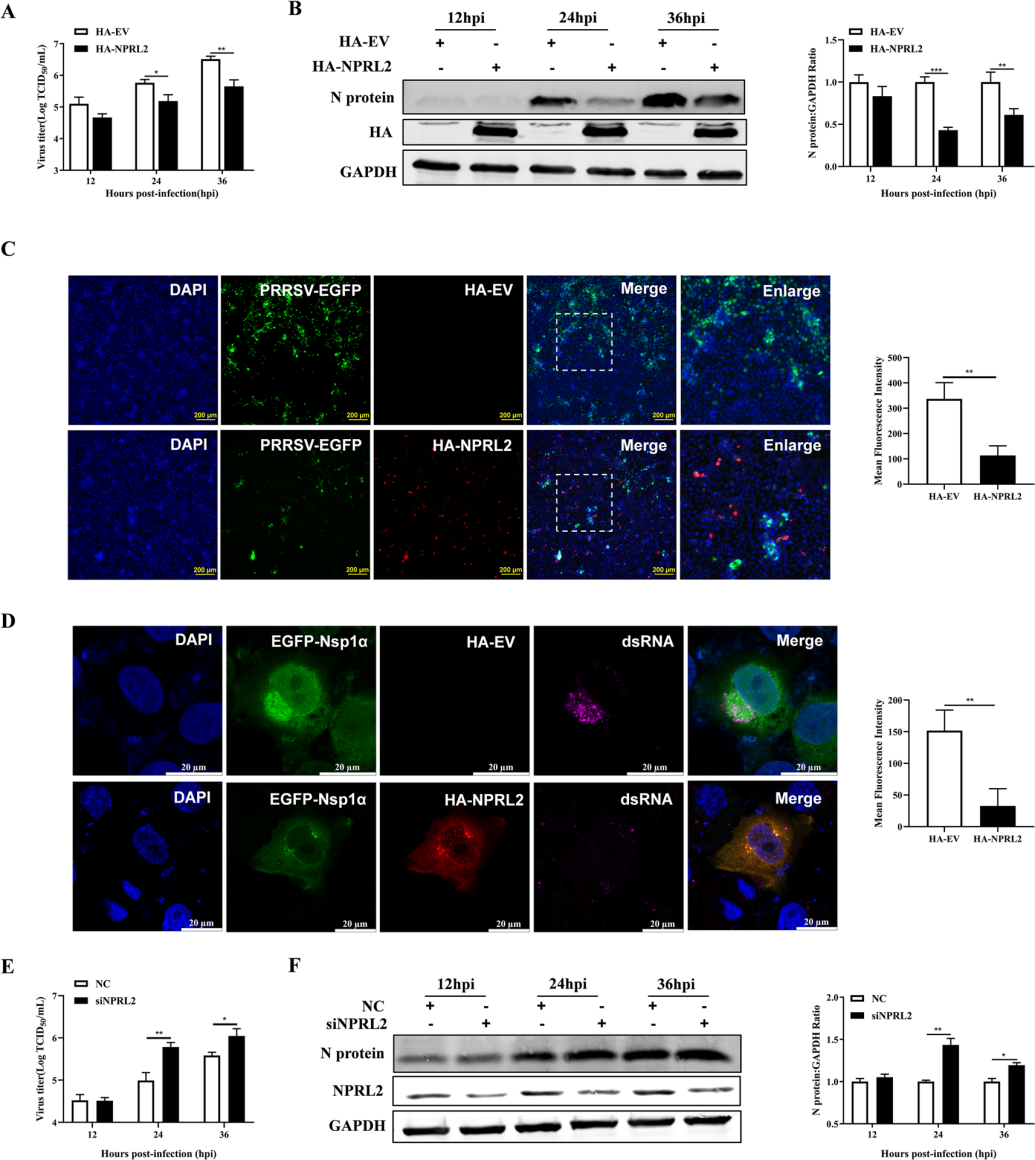
**NPRL2 inhibits PRRSV replication**.** A**, **B** Overexpression of NPRL2 inhibits PRRSV replication. Marc-145 cells transfected with HA-NPRL2 or empty vector (EV) control were infected with PRRSV (MOI of 0.1 TCID_50_ per cell). At the indicated times post-infection, cells and supernatants were harvested for **A** TCID_50_ assay to determine viral titers, and **B** immunoblotting analysis of PRRSV N protein levels with quantification normalized to GAPDH. **C** NPRL2 expression reduces PRRSV infection. Marc-145 cells transfected with HA-NPRL2 or EV were infected with PRRSV-EGFP (MOI of 3 TCID_50_ per cell) for 24 h, then fixed and immunostained for HA (red). Nuclei were stained with DAPI (blue). EGFP fluorescence (green) was quantified to assess infection levels. Scale bar, 200 μm. **D** NPRL2 overexpression impairs viral dsRNA accumulation. Marc‑145 cells coexpressing EGFP‑Nsp1α and HA‑NPRL2 (or EV control) were infected with PRRSV (MOI of 0.1 TCID_50_ per cell) for 24 h. Cells were coimmunostained using an anti‑dsRNA antibody (J2 clone) and an anti‑HA antibody, followed by appropriate secondary antibodies. Nuclei were stained with DAPI. The fluorescence intensity of dsRNA puncta was quantified. Scale bar, 20 μm. **E**, **F** Knockdown of NPRL2 enhances PRRSV replication. Marc-145 cells transfected with NPRL2-targeting siRNA (siNPRL2) or negative control siRNA (NC) were infected with PRRSV (MOI of 0.1 TCID_50_ per cell). **E** Viral titers in supernatants were measured by TCID_50_ assay. **F** PRRSV N protein levels were analyzed by immunoblotting with quantification. Data are mean ± SEM of three independent experiments (*n* = 3). *P*-values were calculated by a two-tailed Student’s *t*-test (* *P* < 0.05; ** *P* < 0.01; *** *P* < 0.001).

To assess the role of NPRL2 in PRRSV replication, Marc-145 cells were transfected with HA-NPRL2 plasmids or empty vector (EV) control for 24 h prior to PRRSV infection. Overexpression of NPRL2 significantly inhibited PRRSV replication, as demonstrated by reduced virus titer and N protein levels at both 24 and 36 h post-infection (hpi) compared with EV-transfected controls (Figures 3A and B). Consistently, the fluorescence intensity of EGFP-labeled PRRSV was markedly weaker in HA-NPRL2-expressing cells (Figure 3C). We next examined the formation of viral dsRNA, a replication intermediate and marker for active replicase complexes. In Marc-145 cells coexpressing EGFP-Nsp1α and HA-NPRL2, dsRNA signals were significantly diminished upon PRRSV infection compared with EV-transfected controls (Figure 3D), indicating that NPRL2 overexpression impairs the RNA-synthesizing activity of the viral replicase complex. To confirm these results, we knocked down NPRL2 expression using specific siRNA. As expected, NPRL2 knockdown in Marc-145 cells increased virus titer and significantly elevated N protein expression (Figures 3E and F).

### NPRL2 facilitated autophagic degradation of PRRSV Nsp1α

**Figure 4:**
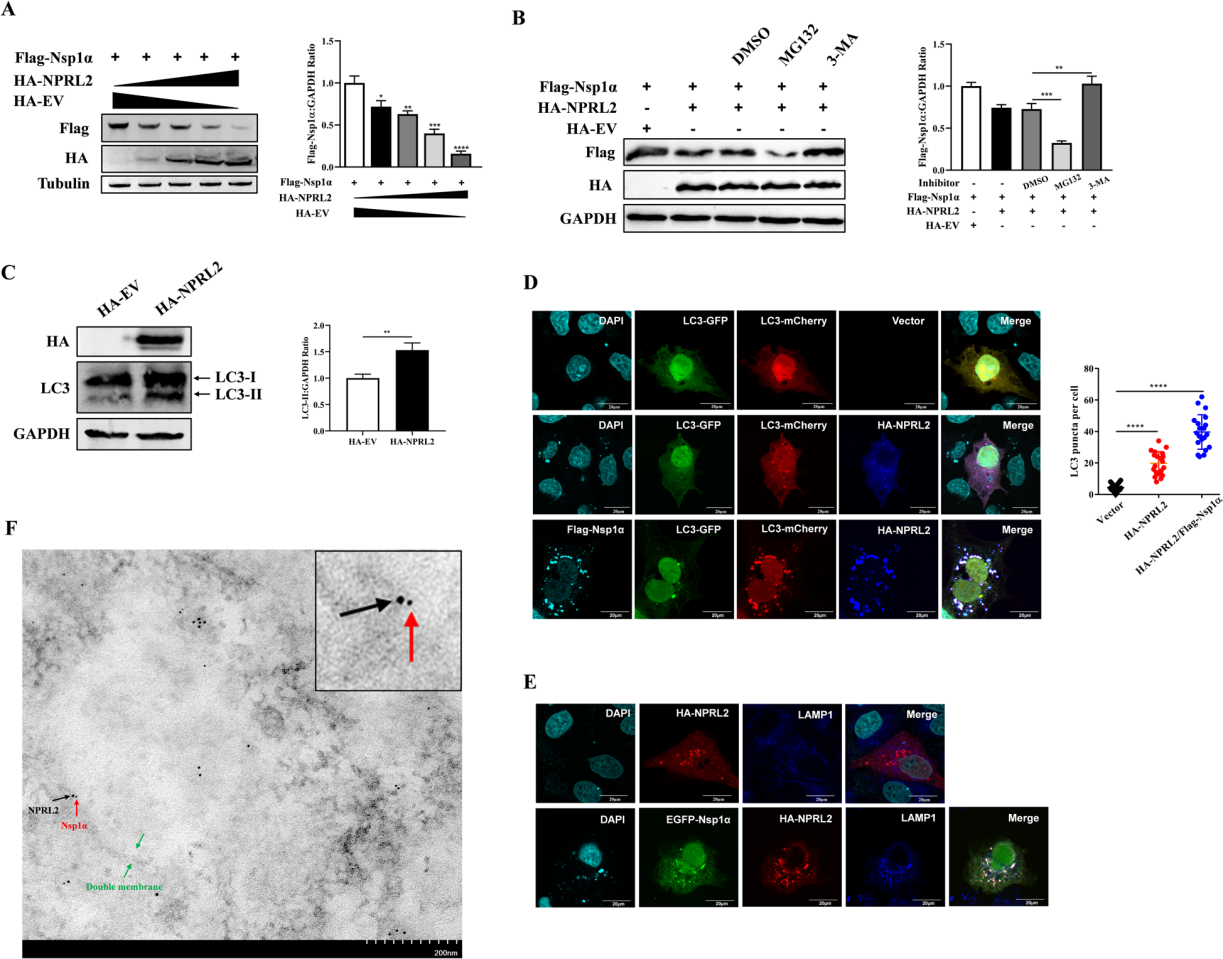
**NPRL2 facilitates autophagic degradation of PRRSV Nsp1α**. **A** NPRL2 reduces Nsp1α levels dose-dependently. Immunoblot analysis of HEK-293 T cells coexpressing Flag-Nsp1α with increasing amounts of HA-NPRL2. Nsp1α levels were quantified and normalized to Tubulin. **B** NPRL2-mediated Nsp1α degradation is autophagy-dependent. HEK-293 T cells coexpressing Flag-Nsp1α and HA-NPRL2 were treated with MG132 (20 μM) or 3-MA (5 mM) for 16 h. Nsp1α levels were analyzed by immunoblotting and quantified. **C** NPRL2 induces autophagic flux. Immunoblot analysis of LC3-I/II conversion in HEK-293 T cells expressing HA-NPRL2. LC3-II levels were quantified and normalized to GAPDH. **D** NPRL2 promotes autophagosome recruitment. Marc-145 cells coexpressing mCherry-GFP-LC3 with Flag-Nsp1α and/or HA-NPRL2 were immunostained with the indicated antibodies. Autolysosomes (mCherry-only puncta) were quantified. Scale bar, 20 μm. **E** NPRL2 and Nsp1α colocalize with lysosomes. Marc-145 cells expressing EGFP-Nsp1α (green) and HA-NPRL2 were immunostained with anti-HA (red) and anti-LAMP1 (blue) antibodies. Scale bar, 20 μm. **F** Immunoelectron microscopy visualizes NPRL2 and Nsp1α on autophagic structures. Marc-145 cells coexpressing HA-NPRL2 and Flag-Nsp1α were analyzed by immunoelectron microscopy using anti-HA (12-nm gold, black arrows) and anti-Flag (4-nm gold, red arrows) antibodies. Autophagic structures are indicated (green arrows). Scale bar, 200 nm. Data are representative of three independent experiments. *P < 0.05, **P < 0.01, ***P < 0.001, ****P < 0.0001 (Student’s *t*-test).

We demonstrated that NPRL2 interacts with Nsp1α and inhibits PRRSV replication. To investigate whether this inhibition occurs through regulation of Nsp1α expression, HEK-293 T cells were cotransfected with increasing concentrations of HA-NPRL2 and a fixed amount of Flag-Nsp1α, followed by protein analysis using western blotting. NPRL2 expression resulted in a significant, dose-dependent reduction in Nsp1α levels (Figure 4A).

As the ubiquitin–proteasome system and autophagy represent the major protein degradation pathways in eukaryotic cells [22–24], we next sought to determine which mechanism mediates NPRL2-induced Nsp1α degradation. HEK-293 T cells expressing both Flag-Nsp1α and HA-NPRL2 were treated with the proteasome inhibitor MG132 or the autophagy inhibitor 3-methyladenine (3-MA). As shown in Figure 4B, 3-MA blocked the NPRL2-mediated degradation of Nsp1α. Notably, MG132 treatment further enhanced Nsp1α degradation. This is consistent with studies showing that proteasome inhibition can compensatory activate autophagy [25]. Thus, the ability of MG132 to facilitate Nsp1α loss underscores the primary role of the autophagy pathway in this process.

Given previous reports that NPRL2 induces autophagy [26–28], we assessed autophagic activation following its expression. Transfection of HEK-293 T cells with HA-NPRL2 enhanced LC3-II expression, indicating increased autophagosome formation (Figure 4C). To evaluate autophagic flux, we utilized the mCherry-GFP-LC3 tandem reporter construct. The GFP signal is quenched in the acidic environment of the lysosome, whereas the mCherry signal remains stable [29]. Cells expressing HA-NPRL2 exhibited increased mCherry-positive puncta compared with empty vector-transfected controls (Figure 4D), indicating that NPRL2 promotes autophagosome-lysosome fusion. Furthermore, Nsp1α colocalized with mCherry-LC3 in NPRL2-expressing cells (Figure 4D). Using LAMP1 as a lysosomal marker, we observed that NPRL2 alone did not colocalize with LAMP1, but coexpression with Nsp1α induced their colocalization (Figure 4E). Together, these findings demonstrate that NPRL2 promotes selective autophagic degradation of Nsp1α.

To further characterize the subcellular localization of NPRL2 and Nsp1α, we performed immunoelectron microscopy (IEM) in Marc-145 cells cotransfected with HA-NPRL2 and Flag-Nsp1α. HA-NPRL2 was labeled with 12-nm colloidal gold particles (Figure 4F, black arrows), and Flag-Nsp1α was detected using 4-nm gold particles (Figure 4F, red arrows). IEM analysis confirmed that NPRL2 and Nsp1α colocalize on autophagosomal membranes (Figure 4F). Collectively, these results demonstrate that NPRL2 induces complete autophagic flux and recruits Nsp1α to autophagosomes for degradation.

### NPRL2 targets the K150 site of Nsp1α for K63 ubiquitination

**Figure 5:**
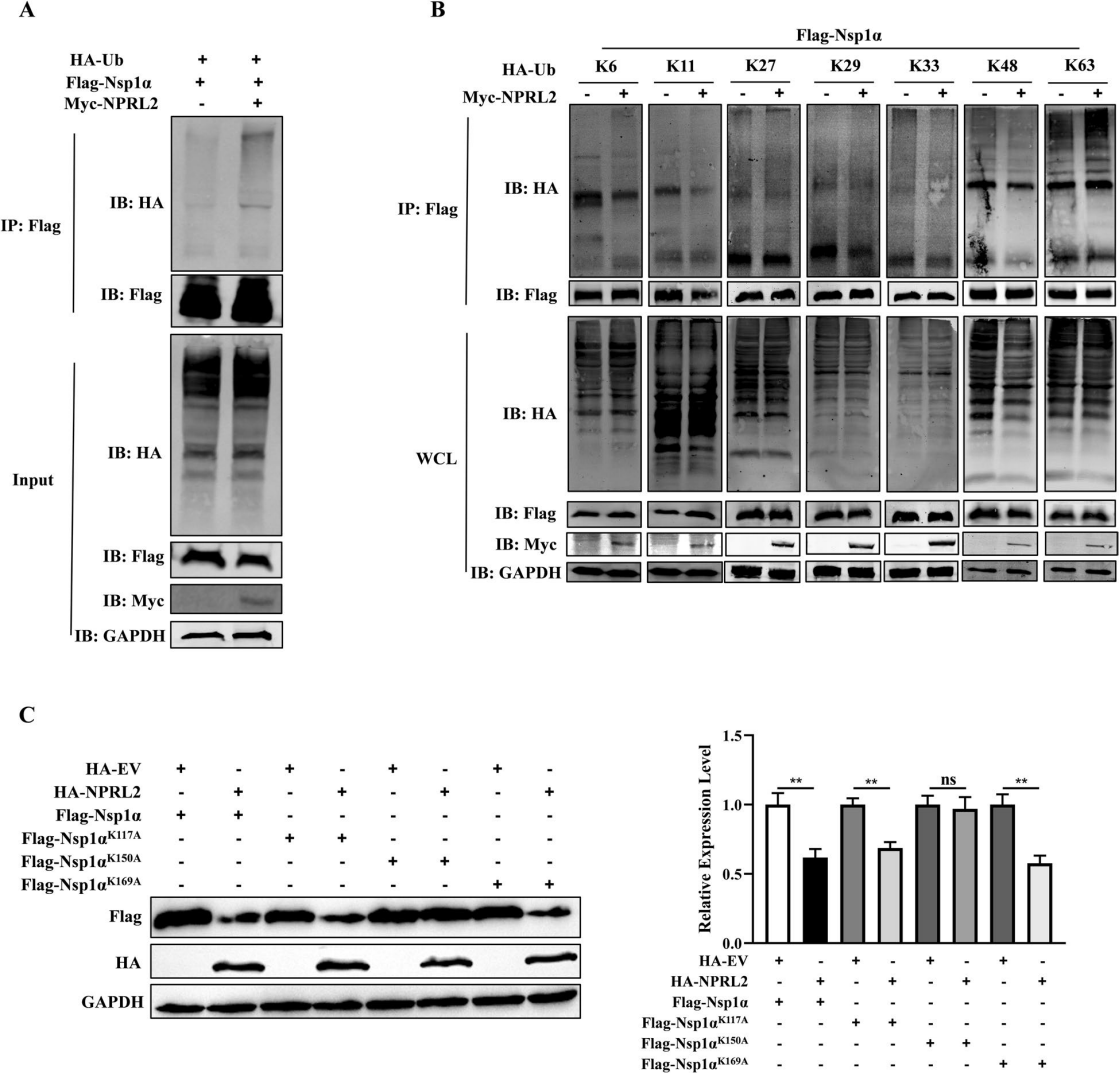
**NPRL2 mediates K63-linked ubiquitination of Nsp1α at K150**.** A**, **B** NPRL2 promotes K63-linked polyubiquitination of Nsp1α. HEK-293 T cells were cotransfected with Flag-Nsp1α, Myc-NPRL2, and either **A** HA-Ubiquitin (WT) or **B** the indicated HA-Ub (K-only) mutants. At 36 h post-transfection, cell lysates were subjected to immunoprecipitation (IP) with anti-Flag antibody, followed by immunoblotting (IB) with antibodies against HA, Flag, and Myc. GAPDH served as a loading control for whole-cell lysates (WCL). **C** The K150 residue is critical for NPRL2-mediated degradation of Nsp1α. HEK-293 T cells were cotransfected with HA-NPRL2 and either wild-type (WT) Flag-Nsp1α or its lysine-to-arginine mutants (K117A, K150A, K169A). Whole-cell lysates were analyzed by IB. Nsp1α protein levels were quantified by densitometry and normalized to GAPDH. Data are representative of three independent experiments. *P < 0.05, **P < 0.01 (Student’s *t*-test).

Selective autophagy relies on cargo receptors that either bind directly to their targets or recognize polyubiquitin chains attached to cargo proteins [30]. We found that NPRL2 significantly enhanced the polyubiquitination of Nsp1α (Figure 5A). Since ubiquitin can form polyubiquitin chains through seven lysine residues (K6, K11, K27, K29, K33, K48, and K63) [31], we cotransfected HEK-293 T cells with NPRL2, Nsp1α and each of the seven ubiquitin mutants. Coimmunoprecipitation assays revealed that NPRL2 specifically promoted K63-linked polyubiquitination of Nsp1α (Figure 5B).

Ubiquitination involves the covalent attachment of ubiquitin to lysine residues on substrate proteins [32]. To identify the critical lysine residue targeted by NPRL2, we generated individual alanine substitutions of all three lysine sites (K117, K150, K169) in Nsp1α and expressed these mutants with NPRL2 in HEK-293 T cells. Notably, the K150A mutation nearly abolished NPRL2-mediated degradation of Nsp1α, while the other mutants had no significant effect (Figure 5C). Together, these results demonstrate that NPRL2 selectively targets Nsp1α for degradation through K63-linked polyubiquitination at the K150 site.

## Discussion

**Figure 6:**
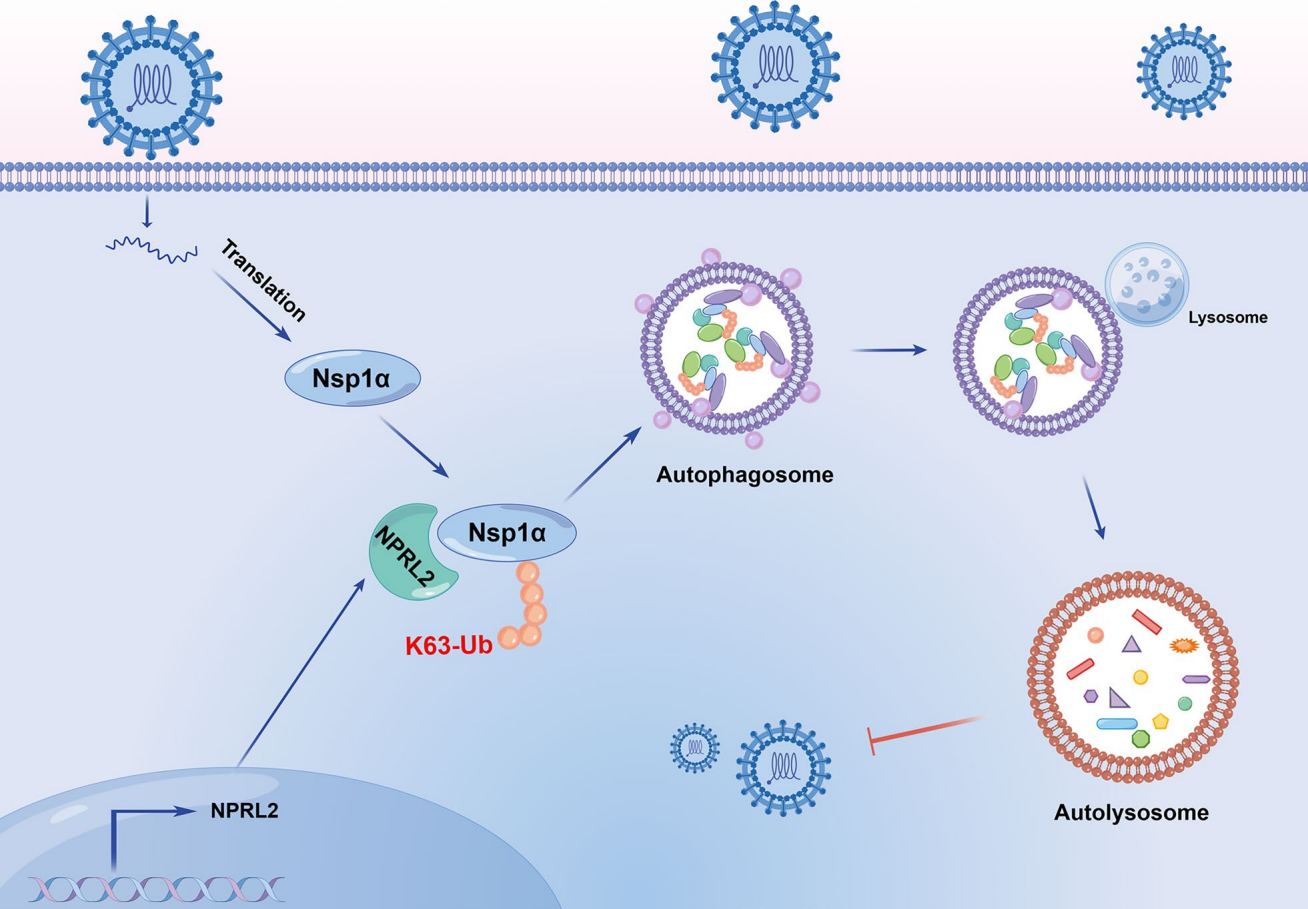
**Working model of the antiviral mechanism of NPRL2**. NPRL2 counteracts PRRSV infection by orchestrating K63-linked ubiquitination of the viral protein Nsp1α at K150, which triggers its degradation through the autophagy-lysosome pathway, thereby restoring host antiviral defense and suppressing viral replication.

This study identifies a novel antiviral role for NPRL2, an emerging regulator of protein degradation, demonstrating the molecular mechanism by which it acts as a novel restriction factor against PRRSV infection. We discovered that NPRL2 employs a “tag-and-degrade” strategy: it specifically mediates K63-linked ubiquitination of the viral protein Nsp1α at lysine 150, thereby directing its degradation through the autophagy–lysosomal pathway (Figure 6). This finding not only expands our understanding of the mechanisms of action of host restriction factors but also provides new targets for antiviral therapy.

At the mechanistic level, this study elucidates the precise pathway through which NPRL2 regulates protein degradation. Unlike the typical K48-linked ubiquitination that targets substrates for proteasomal degradation [33, 34], NPRL2 specifically promotes K63-linked ubiquitination of Nsp1α. This finding aligns with the “ubiquitin code” hypothesis, which posits that different ubiquitin chain linkages determine distinct fates of substrate proteins [35]. We precisely mapped the ubiquitination site to the lysine 150 (K150) residue of Nsp1α through site-directed mutagenesis. Notably, the K150 residue is located within the PCPα domain of Nsp1α, which is not only critical for its interaction with NPRL2 but also essential for its proteolytic activity [36–38]. Therefore, K63-linked ubiquitination at this site likely serves a dual purpose: it flags the protein for autophagic destruction and may simultaneously interfere with the intrinsic function of the PCPα domain, thereby delivering a more potent blow to the virus. This discovery provides a new perspective for understanding the specificity of protein degradation and offers a precise target for developing inhibitors against this site.

Critically, the autophagic degradation of Nsp1α directly underpins NPRL2’s antiviral activity. As Nsp1α is a key viral virulence factor essential for PRRSV replication and immune evasion [39–41], its targeted removal by NPRL2 would be expected to severely compromise viral fitness, which is consistent with our observed reduction in viral titers and N protein expression upon NPRL2 overexpression.

PRRSV Nsp1α contains a papain-like protease domain (PLPα), which is essential for viral polyprotein processing and immune evasion [38, 42]. It is therefore reasonable to consider whether PLPα possesses intrinsic self‑degradation activity that could contribute to the turnover of Nsp1α independently of host factors. However, several lines of evidence argue against this possibility under our experimental conditions. First, in the absence of NPRL2, Nsp1α protein levels remained stable throughout the time course of our assays, indicating that any potential autocatalytic cleavage—if it occurs—is not a major determinant of Nsp1α stability in this context. Second, the degradation we observed was strictly dependent on NPRL2 expression and was associated with K63‑linked ubiquitination at a specific lysine residue (K150), highlighting a regulated, host‑directed process rather than a constitutive viral self‑cleavage mechanism. Third, while PLPα exhibits deubiquitinating and delSGylating activities that modulate host immune responses [9, 10], its established proteolytic function is trans-cleavage at defined polyprotein junctions and is not known to mediate autoproteolytic degradation. Therefore, the observed reduction in Nsp1α is most likely driven by the host pathway.

Our study reveals a novel function for NPRL2 in antiviral defense through its dynamic, signal-dependent recruitment to lysosomes. Using the mCherry-GFP-LC3 dual-labeling system, we demonstrated that NPRL2 promotes autophagic flux and autophagosome-lysosome fusion (Figure 4D). Particularly noteworthy was our observation that the recruitment of NPRL2 to LAMP1-positive lysosomes was only detectable upon Nsp1α overexpression and was virtually undetectable under normal conditions (Figure 4E). This finding strongly suggests that NPRL2 is not a static lysosomal component but is instead specifically recruited to the site of degradation upon engagement with its viral substrate.

This discovery establishes NPRL2 as a conditionally recruited adaptor. In the absence of viral infection, NPRL2 exhibits a diffuse localization. However, the presence of Nsp1α acts as a potent molecular signal that triggers its translocation, ensuring that the autophagic-lysosomal degradation machinery is engaged only when needed. This substrate-induced recruitment mechanism mirrors strategies employed by other innate immune sensors. For instance, the activation of many pattern-recognition receptors (PRRs) also requires ligand binding, ensuring a targeted and controlled immune response [43–45]. Similarly, NPRL2 operates as a precise sensor for a specific viral antigen, unleashing a potent degradation machinery only upon encounter with its target, thereby minimizing unnecessary cellular energy expenditure and potential off-target effects.

The scientific and translational significance of this study is reflected in providing new insights for “host-directed therapy” [46]. Traditional antiviral strategies mostly target viral proteins directly, but the high mutation rate of viruses often leads to drug resistance [47–49]. In contrast, targeting the host-mediated degradation of viral proteins may present a strategy less prone to such resistance. Our mechanistic elucidation of the NPRL2–Nsp1α axis suggests concrete translational avenues. The identification of the critical K150 ubiquitination site within Nsp1α provides a precise target for structure-based drug design. Small-molecule inhibitors that block this site could stabilize Nsp1α and may be explored as research tools or as lead compounds for developing antiviral therapeutics. Furthermore, the mechanism by which NPRL2 acts as a natural adaptor, bridging a viral protein to the autophagic machinery, is conceptually analogous to proteolysis-targeting chimera (PROTAC) technology [50, 51]. This insight opens the possibility of designing synthetic PRRSV-specific PROTACs to recruit Nsp1α for degradation, representing a novel and highly specific antiviral modality. While further validation in animal models is essential, enhancing the NPRL2-mediated defense pathway represents a promising host-centric strategy that could complement existing vaccines and biosecurity measures in the integrated management of PRRS.

However, this study also has some limitations. First, whether NPRL2 directly participates in the ubiquitin transfer process or functions by recruiting other E3 ligases remains to be clarified. Second, the expression levels of NPRL2 in different cell types and tissues and their effects on antiviral efficacy need systematic evaluation. Finally, validating NPRL2’s antiviral effect in animal models will be a crucial step toward clinical translation. It is worth noting that viruses may have evolved corresponding countermeasures. Future research could explore whether PRRSV inhibits NPRL2 function through certain mechanisms and whether the host has other backup mechanisms to counter viral escape.

In conclusion, our work identifies NPRL2 as a central player in a newly elucidated antiviral pathway, which bridges the detection of a viral virulence factor to its elimination via selective autophagy. Targeting this NPRL2–Nsp1α axis presents a promising host-directed therapeutic strategy not only against PRRSV, a major pathogen plaguing the swine industry, but also potentially against other viruses that may be susceptible to similar targeted degradation mechanisms.

## Data Availability

No datasets were generated or analyzed during the current study.
